# Supplementary material for: Exact Hybrid Particle/Population Simulation of Rule-Based Models of Biochemical Systems
Source: PLoS Comput Biol. 2014 Apr 3;10(4):e1003544. doi: 10.1371/journal.pcbi.1003544 (PMC3974646; doi:10.1371/journal.pcbi.1003544)
Supplement: Text S1 — Sec. S1: Details of the monetary cost analysis shown in Fig. 11; Sec. S2: Space complexity analyses for the network-based SSA, network-free, HPP, and hypothetical aHPP methods (Fig. 10); Sec. S3: Overview of BNGL, model files, and running HPP simulations with BioNetGen/NFsim; Sec. S4: BNGL formalism and the formal foundation of the PNE algorithm (with pseudocode). (PDF) [file pcbi.1003544.s003.pdf]

Exact hybrid particle/population simulation of rule-based models of biochemical systems

Supporting Information: Text S1

Justin S. Hogg[*], Leonard A. Harris[†], Lori J. Stover, Niketh S. Nair, and James R. Faeder[‡]
Department of Computational and Systems Biology,
University of Pittsburgh School of Medicine, Pittsburgh, PA 15260

Contents

S1 Monetary cost analysis **2**

S2 Space complexity and automated HPP **3**
S2.1 Network-based methods . . . 3
S2.2 Network-free methods . . . 4
S2.3 Hybrid particle/population . . . 4
S2.4 Optimal HPP . . . 4
S2.4.1 Small volumes . . . 4
S2.4.2 Large volumes . . . 5
S2.4.3 Intermediate volumes . . . 5

S3 Rule-based modeling with BioNetGen **6**
S3.1 Basic elements of a BNGL model . . . 7
S3.1.1 Macromolecules as structured objects . . . 7
S3.1.2 Molecular complexes as hierarchical graphs . . . 7
S3.1.3 Patterns as subgraphs . . . 8
S3.1.4 Reaction rules as graph transformations . . . 8
S3.2 Anatomy of a BNGL file . . . 8
S3.3 Generating and simulating an HPP model with BioNetGen/NFsim . . . 9

S4 BioNetGen graph formalism and the partial network expansion algorithm **10**
S4.1 Graph-theoretic BNGL . . . 10
S4.1.1 Pattern graphs . . . 10
S4.1.2 Complex graphs and ensembles of complexes . . . 11
S4.1.3 Pattern graph isomorphism and species . . . 11
S4.1.4 Embeddings of pattern graphs . . . 12
S4.1.5 Reaction rules . . . 13
S4.1.6 BNGL models . . . 16
S4.2 Formal basis for the PNE algorithm . . . 16
S4.2.1 Hybrid ensembles . . . 16
S4.2.2 Child rules and rule restriction . . . 17
S4.2.3 Population-mapping rules . . . 18
S4.2.4 The PNE algorithm . . . 18

[*]justinshogg@gmail.com
[†]lh64@cornell.edu
[‡]faeder@pitt.edu

S1 Monetary cost analysis

We assume that each simulation is run on one virtual core (with possibly more than one simulation running per core) and there is no memory sharing among simulations. Let $\mathcal{I}$ be an Amazon EC2 instance type, $m_{\mathcal{I}}$ the total instance memory, $v_{\mathcal{I}}$ the number of virtual cores in the instance, $u_{v_{\mathcal{I}}}$ the number of compute units per virtual core, $C_{\mathcal{I}}$ the cost of the instance per unit time, and N the number of simultaneous simulations running on an instance. For each configuration pair $(\mathcal{I}, N)$, with $N \in \{1, 2, \ldots\}$, we will compute the cost (per unit time) per "effective" compute unit (ECU). We will then obtain the minimum cost by finding that (those) configuration(s) (multiple instances may have the same cost) with the lowest cost *under the constraint* that the instance(s) has (have) sufficient memory to run N simulations.

Under our assumption that a simulation is run on one virtual core, the average number of compute units available per simulation is the minimum of the number of compute units per core and the total number of compute units divided by the number of running simulations,

$$u_s(\mathcal{I}, N) = u_{v_{\mathcal{I}}} \cdot \min\{1, v_{\mathcal{I}}/N\}.$$

The number of ECUs is the number of running simulations multiplied by the average number of compute units per simulation,

$$\epsilon(\mathcal{I}, N) = N \cdot u_s(\mathcal{I}, N) = u_{v_{\mathcal{I}}} \cdot \min\{N, v_{\mathcal{I}}\}.$$

Note that the number of ECUs is equal to the total number of compute units if $N \geq v_{\mathcal{I}}$ and less if $N < v_{\mathcal{I}}$. The intuition here is that if the memory required per simulation limits the number of simulations that can be run simultaneously on an instance to fewer than the number of virtual cores, then we must treat the instance as if it had fewer virtual cores since some cannot be utilized.

The cost (per unit time) per ECU for a given configuration is then the cost of the instance divided by the number of ECUs,

$$C(\mathcal{I}, N) = \frac{C_{\mathcal{I}}}{\epsilon(\mathcal{I}, N)} = \frac{C_{\mathcal{I}}}{u_{v_{\mathcal{I}}} \cdot \min\{N, v_{\mathcal{I}}\}}.$$

For a given configuration, the memory available per simulation is

$$m(\mathcal{I}, N) = m_{\mathcal{I}}/N.$$

Assuming that each simulation run requires m_s units of memory, the minimum cost per ECU is obtained by finding the lowest configuration cost under the constraint that the configuration has sufficient memory available,

$$C_{\min}(m_s) = \min_{\text{all } (\mathcal{I},N)} \{C(\mathcal{I}, N) : m_s \leq m(\mathcal{I}, N)\}.$$

We need only consider $N \leq v_{\mathcal{I}}$ when searching for the minimum cost because $\epsilon(\mathcal{I}, N)$ is constant ($= u_{v_{\mathcal{I}}} v_{\mathcal{I}}$) for $N \geq v_{\mathcal{I}}$. $C_{\min}(m_s)$ has been calculated considering all Amazon EC2 *Standard*, *High-CPU*, and *High-Memory* instance types [1] and plotted in Fig. 8 of the main text. The configuration(s) associated with the minimum cost is (are) simply the set

$$\chi(m_s) = \{(\mathcal{I}, N) : C(\mathcal{I}, N) = C_{\min}(m_s) \wedge m_s \leq m(\mathcal{I}, N)\}.$$

Example: The Amazon EC2 *High-Memory Double Extra Large* instance has four virtual cores with 3.25 compute units each, 34.2 GB of total memory, and a cost of \$1.00/hr (January 2012 on-demand pricing, Linux operating system) [1]:

$$\begin{aligned} v_{\mathcal{I}} &= 4, \\ u_{v_{\mathcal{I}}} &= 3.25, \\ m_{\mathcal{I}} &= 34.2, \\ C_{\mathcal{I}} &= 1.00. \end{aligned}$$

The number of compute units available per simulation is

$$u_s(\mathcal{I}, N) = 3.25 \cdot \min\{1, 4/N\},$$

Figure S1.1: Minimum effective cost for the Amazon EC2 *High-Memory Double Extra Large* instance as a function of required memory per simulation run.

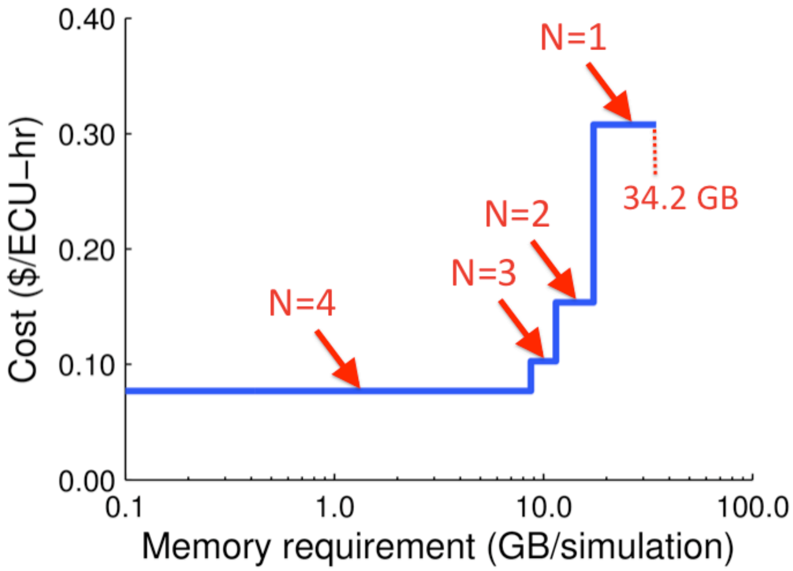

and the number of ECUs is

$$\epsilon(\mathcal{I}, N) = 3.25 \cdot \min\{N, 4\}.$$

This gives a cost per ECU of

$$C(\mathcal{I}, N) = \frac{1.00}{3.25 \cdot \min\{N, 4\}},$$

with memory availability per simulation of

$$m(\mathcal{I}, N) = 34.2/N.$$

For this instance type, we show in Fig. S1 the minimum effective cost, $C^{\mathcal{I}}_{\text{min}}(m_s)$, versus memory per simulation, m_s. Up to $m_s = 8.6$ GB it is possible to run four simultaneous simulations at a cost of $\sim\$0.08$/ECU-hr. We can run three simulations at $\sim\$0.10$/ECU-hr for $8.6 < m_s \leq 11.4$ GB, two simulations at $\sim\$0.15$/ECU-hr for $11.4 < m_s \leq 17.1$ GB, and one simulation at $\sim\$0.31$/ECU-hr for $17.1 < m_s \leq 34.2$ GB. Above $m_s = 34.2$ GB we obviously cannot run any simulations because the memory requirement exceeds the total memory available in the instance.

S2 Space complexity and automated HPP

S2.1 Network-based methods

In network-based stochastic simulation methods, the system state is represented by a species population vector and the reaction network by a list of reactions [stoichiometry vector, rate law, and current propensity (rate)] along with a reaction dependency tree for efficient propensity updates [2]. Keeping species concentrations fixed, when the reactor volume is increased the population counters and reaction propensities all increase, but the space required to store the state information remains constant. This is one of the beneficial properties of network-based methods. However, in practice it is possible for this constant cost to be prohibitively large if the reaction network is combinatorially large (e.g., infinite), such as arises naturally in models of multimeric protein complex assembly and branching polymers [3, 4].

S2.2 Network-free methods

In contrast to network-based methods, the memory cost of network-free (NF) methods scales approximately linearly with reactor volume (with fixed concentrations). The memory required for a NF simulation consists of two quantities: the fixed simulator memory and the particle-dependent memory. The former is the memory required to initialize the NF simulator with zero particles. This is just a constant value, $\overline{a}_{\mathrm{nf}}$, which may depend on the software implementation. The latter can be calculated by summing over the memory cost of populating each species. Assuming stochastic fluctuations are small compared to mean concentrations, the population of a species is approximately the product of a volume-independent concentration and the reactor volume. Hence, the cost of representing a species is the product of a species-specific constant, $b_{\mathrm{nf}}(s)$, the species concentration, $[s]$, and the reactor volume, V. The species-specific constant accounts for the cost of representing the molecular structure and all reactant pattern matches for one instance of the species. The total memory cost of the particles is obtained by summing over the cost of all species in the set of reachable species S. Since each term in the sum is linearly dependent on volume, the sum is also linear in volume. At large volumes the constant cost of the NF simulator becomes negligible. Thus, we obtain a linear memory cost in terms of volume, i.e.,

$$M_{\mathrm{nf}}(V) = \overline{a}_{\mathrm{nf}} + B_{\mathrm{nf}} \cdot V = O(V) \text{ as } V \to \infty,$$

with $B_{\mathrm{nf}} \equiv \sum_{s\in S}[s] \cdot b_{\mathrm{nf}}(s)$.

S2.3 Hybrid particle/population

The memory cost of HPP is also linear with respect to reactor volume and we can derive it in the same way as for NF. The cost of initializing the simulator with zero particles, $a_{\mathrm{hpp}}(P)$, includes the constant cost of initializing both the simulator and the partial reaction network for a specific set of population species $P \subset S$. The particle cost term has the same form as for NF except that we only sum over the non-population species, $S \backslash P$. Thus, we have

$$M_{\mathrm{hpp}}(V, P) = a_{\mathrm{hpp}}(P) + B_{\mathrm{hpp}}(P) \cdot V = O(V) \text{ as } V \to \infty,$$

with $B_{\mathrm{hpp}}(P) \equiv \sum_{s\in S\backslash P}[s] \cdot b_{\mathrm{hpp}}(s, P)$.

Note that the species-specific memory constant, $b_{\mathrm{hpp}}(s, P)$, may be different than that for NF, $b_{\mathrm{nf}}(s)$. This is because of the memory required in HPP to match the particle-based species to the mixed particle-population and population-mapping rules. In general, $b_{\mathrm{nf}}(s) \leq b_{\mathrm{hpp}}(s, P)$. It is also typically the case that the fixed simulator cost of HPP, $a_{\mathrm{hpp}}(P)$, is greater than that for NF, $\overline{a}_{\mathrm{nf}}$. Thus, in order to reduce memory use the set of population species, P, must be chosen such that $B_{\mathrm{hpp}}(P) < B_{\mathrm{nf}}$. It is difficult to calculate these quantities explicitly, but we can see the factors that contribute to a "good" set of population species, i.e., high species concentrations and participation in a small number of mixed particle-population rules.

S2.4 Optimal HPP

With a formula for HPP memory cost, we can now imagine a hypothetical algorithm where an optimal set of population species is chosen "on the fly" to minimize memory cost and the hybrid model is adapted accordingly. Let us call this method "automated HPP" (aHPP). How would the memory use of aHPP compare to HPP, NF, and SSA? We will begin to answer this question by considering the behavior at small and large volumes. We will then scrutinize the intermediate region between these two extremes.

S2.4.1 Small volumes

For fixed species concentrations, as the reactor volume approaches zero the populations of all species also approach zero. There is little benefit, therefore, to lumping populations in this case since the memory cost associated with representing the partial reaction network will outweigh any savings due to lumping. Thus, at very small volumes aHPP will treat all species as particles (as in NF methods) and, consequently, the memory scaling will be linear with volume.

S2.4.2 Large volumes

Memory scaling of aHPP at large volumes will depend on whether the model encodes a finite or countably-infinite network. For a finite network, the population of every reachable species will become large as the volume increases towards infinity. At some point, the cost of representing species as particles (linear cost) will exceed that of representing the entire reaction network (constant cost). Thus, at very large volumes aHPP will treat every species as a population and, consequently, the memory scaling will be constant with volume.

The case of countably-infinite networks is more difficult. In general, the memory scaling with volume will depend on the specifics of the model, with the best-case scenario being nearly constant and the worst-case being linear. An example of the worst case is the TLBR model in the gel phase [3, 4], where a "super aggregate" forms that subsumes the majority of the molecules in the system. Increasing the volume only increases the size of the aggregate, so little to no lumping is possible. aHPP will operate essentially as an NF method in this case and, consequently, memory use with scale linearly with volume.

The situation will not be so dire in all cases, however. For example, consider a system with N particles, where each particle may take on a countably infinite number of states $s \in \{1, 2, 3, \ldots\}$ and has independent probability $p(s) = 0.5^s$ of being in state s. The probability that none of the particles are in state s is thus $p(N_s = 0) = (1 - 0.5^s)^N$, where N_s is the number of particles in state s. If we consider each state to be an independent *species*, then we can estimate the expected number of populated species, ϵ, by summing over the probability that each state is populated:

$$\epsilon = \sum_{s=1}^{\infty} (1 - p(N_s = 0)) = \sum_{s=1}^{\infty} \left(1 - (1 - 0.5^s)^N\right) = O(\log N) \text{ as } N \to \infty. \quad (1)$$

We see in Fig. S2 that, in the limit of large N, this sum grows logarithmically with N. The intuition here is that an increase in the number of particles (increasing the volume while holding concentrations fixed) results in larger populations of previously populated species (those with small s) and new instances of rare species that were not previously populated (those with large s). However, the rate of accumulation of novel species diminishes as the volume gets large, leading to logarithmic growth.

This is clearly an artificial example in that we are not considering intermolecular binding or the mechanisms of species formation. However, it does provide a simple example of sub-linear memory scaling for an infinite-network model. Obviously, it is difficult to say how the memory cost will scale for more realistic infinite-network models, but if the bulk of the population resides in relatively few of the species, with the remainder of the species being increasingly rare, then we expect that "something like" logarithmic scaling will be achievable in many cases.

S2.4.3 Intermediate volumes

Finally, we consider volumes between "very large" and "very small." Let us denote the minimal memory use function by $m^* : V \to (0, \infty)$. We will say that $P_V \subseteq S$ is the optimal lumping for volume V if it achieves the minimum memory use. We will argue that m^* is monotonic increasing and concave with respect to V. This yields a qualitative picture for aHPP where memory use scales linearly for very small volumes, is monotonic increasing and concave for intermediate volumes, and is somewhere between constant (finite networks and best case for infinite networks) and linear (worst case for infinite networks) at very large volumes. This behavior, along with those for NF, HPP, and network-based SSA, is sketched in Fig. 9 of the main text.

First, suppose $0 < V_0 < V_1$ and let P_1 be the optimal lumping for V_1. Since $B_{\text{hpp}}(P_1) \geq 0$, we have

$$\begin{aligned} m^*(V_0) &\leq M_{\text{hpp}}(V_0, P_1) = a_{\text{hpp}}(P_1) + B_{\text{hpp}}(P_1) \cdot V_0 \\ &\leq a_{\text{hpp}}(P_1) + B_{\text{hpp}}(P_1) \cdot V_1 = M_{\text{hpp}}(V_1, P_1) = m^*(V_1). \end{aligned}$$

Thus, m^* is monotonic increasing with volume.

Now, suppose $0 < V_0 < V_1$ and $0 \leq t \leq 1$. Let the set of population species, P_t, be the optimal lumping for volume $V_t = tV_0 + (1-t)V_1$. Since $M_{\text{hpp}}(V, P_t)$ is linear in V (Sec. S2.3), then by the definition of m^*

Figure S1.2: The sum in Eq. 1 scales approximately logarithmically in the number of particles N as $N \to \infty$.

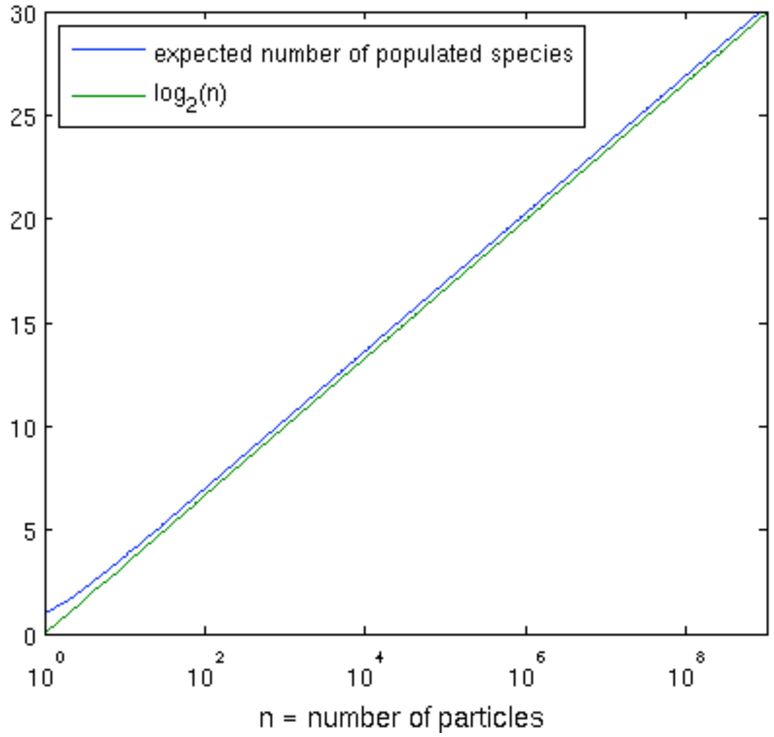

as the minimum memory we must have

$$\begin{aligned} m^*(V_0) \leq M_{\text{hpp}}(V_0, P_t) &= a_{\text{hpp}}(P_t) + B_{\text{hpp}}(P_t) \cdot V_0 \\ &= a_{\text{hpp}}(P_t) + B_{\text{hpp}}(P_t) \cdot V_t - B_{\text{hpp}}(P_t)(V_t - V_0) \\ &= m^*(V_t) - B_{\text{hpp}}(P_t) \cdot (V_t - V_0). \end{aligned}$$

Similarly,

$$m^*(V_1) \leq m^*(V_t) + B_{\text{hpp}}(P_t)(V_1 - V_t).$$

Together these inequalities imply that

$$t\, m^*(V_0) + (1-t)\, m^*(V_1) \leq t\, [\, m^*(V_t) - B_{\text{hpp}}(P_t)(V_t - V_0)\,] + (1-t)\, [\, m^*(V_t) + B_{\text{hpp}}(P_t)(V_1 - V_t)\,]\,.$$

After a bit of algebra, we obtain

$$t\, m^*(V_0) + (1-t)\, m^*(V_1) \leq m^*(V_t) + B_{\text{hpp}}(P_t)\, [\, tV_0 + (1-t)V_1 - V_t\,]\,.$$

Recalling that $V_t = tV_0 + (1-t)V_1$, we obtain the concave relationship

$$t\, m^*(V_0) + (1-t)\, m^*(V_1) \leq m^*\, [\, tV_0 + (1-t)V_1\,]\,.$$

S3 Rule-based modeling with BioNetGen

BioNetGen is a collection of open-source software tools for specifying, simulating, and analyzing rule-based models of complex biochemical systems [5–8]. Models are specified in the BioNetGen language (BNGL), a formal text-based modeling language designed specifically to represent proteins and protein-protein interactions. BNGL is similar to other rule-based languages, notably Kappa [9,10]. In this section, we first provide

a summary of the BNGL syntax. We then discuss the structure of a BNGL input file, including the model specification and a summary of actions that can be applied to a model. We follow with instructions on how to generate an HPP model within BioNetGen and simulate it using NFsim. For further information regarding the syntax and semantics of BNGL, we refer the reader to Refs. [6, 7, 11].

S3.1 Basic elements of a BNGL model

S3.1.1 Macromolecules as structured objects

The fundamental object in BNGL is the *molecule*. Molecules are structured objects that contain *components*. Components have state values (possibly null) and serve as sites for bond formation. Typically, molecules correspond to biological macromolecules such as proteins, while components represent protein functional domains. Component states may describe post-translational modifications, such as phosphorylation, or other properties of the macromolecule, such as conformational state.

Molecules are defined through *molecule type* templates that specify the set of components, and the possible state values of those components, that comprise a molecule. For example, in Blinov et al. [12] the molecule type definition for the epidermal growth factor receptor (EGFR) is

`Egfr(l,r,Y1068~Y~pY,Y1148~Y~pY).`

This specifies that `Egfr` molecules have four components: `l`, `r`, `Y1068`, and `Y1148`. The first two represent a ligand binding domain and a receptor dimerization domain, respectively. The other two represent tyrosine residues 1068 and 1148, which are sites of phosphorylation [13, 14]. Both `Y1068` and `Y1148` can exist in two possible states, `Y` (tyrosine) and `pY` (phosphorylated tyrosine). Names of molecule types, components, and states may be assigned any arbitrary string of alphanumeric characters [6, 7], but conventionally are chosen to correspond to biological entities.

Molecules can be thought of as *instances* of molecule types, the difference being that in molecules a state for each molecular component must be explicitly specified. For example,

`Egfr(l,r,Y1068~pY,Y1148~Y)`

specifies an unligated `Egfr` monomer with a phosphorylated tyrosine at residue 1068 and an unphosphorylated tyrosine at residue 1148.

S3.1.2 Molecular complexes as hierarchical graphs

BNGL represents molecular complexes within a hierarchical graph formalism: components are represented by nodes, molecules are groupings of nodes, and bonds are edges between components [15, 16]. Molecules that are connected by bonds between their components are said to belong to the same *complex*. Bound components are designated by a shared `!LABEL` token following each component in the bond. For example,

`Egf(r!0).Egfr(l!0,r!1,Y1068~pY,Y1148~Y).Egfr(l,r!1,Y1068~pY,Y1148~Y)`

specifies a complex comprised of an `Egfr` dimer and a bound `Egf` ligand. The `Egf` ligand is bound to the first `Egfr` molecule through a bond, labeled '`0`', between its `r` component and the `l` component of the `Egfr` molecule. The two `Egfr` molecules are bound to each other through a second bond, labeled '`1`', between their `r` components. The '`.`' symbol specifies that two molecules are part of the same complex. Molecules that are bound to each other are necessarily in the same complex but molecules in the same complex need not be bound to each other (e.g., the `Egf` ligand and the second `Egfr` molecule).

Two complexes that have the same molecular composition, with the same configuration of bonds and states, are said to be instances of the same *species*. More formally, two complexes are of the same species if their graphs are identical up to a labeled isomorphism [15].

S3.1.3 Patterns as subgraphs

Patterns describe structural motifs that may be found within complexes. In BNGL, patterns are written just like complexes except that components, states, bonds, and molecules that are not part of the motif are omitted. A pattern is said to *match* a complex (or species) if the motif is found within the complex. Note that it is possible for a pattern to match at multiple places within the same complex. In terms of graphs, a pattern is a *subgraph* and a match is a *subgraph isomorphism* from the pattern into the complex graph [5,15]. As an example, the pattern `Egfr(l,r)` matches each instance of `Egfr` with unbound `l` and `r` components, including

`Egfr(l,r,Y1068~Y,Y1148~pY)`,

`Egfr(l,r,Y1068~pY,Y1148~pY)`, and

`Egfr(l,r,Y1068~pY!0,Y1148~pY).Grb2(SH2!0,SH3)`,

where the matches have been underlined. Since the components `Y1068` and `Y1148` are omitted from the pattern they can be in any of their allowed states and either bound or unbound.

A pattern may also require that a component be bound without specifying its binding partner by substituting the bond label with the '+' wildcard (e.g., `Egfr(l!+,r)`). Similarly, the bonding state can be specified as irrelevant (either bound or unbound) by using the '?' wildcard (e.g., `Egfr(l!?,r)`). Finally, if a component is listed but no state is indicated then the pattern will match the component regardless of state.

Patterns have two primary purposes in BNGL [6,7]: (i) they specify parts of a complex that are involved in a reaction; (ii) they define the observable outputs (or simply *observables*) of a model. The second function is critical for facilitating comparisons between simulation predictions and experimental observations.

S3.1.4 Reaction rules as graph transformations

Reaction rules (or simply *rules*) describe classes of reactions that transform the states of complexes in a system. A rule consists of a set of reactant patterns, a set of product patterns, a directionality arrow (unidirectional '->' or bidirectional '<->'), and a rate law. The reactant patterns match reaction sites (motifs) within complexes in the system. The product patterns indicate how the reactant patterns are modified when the rule fires. The rate law determines the firing rate per set of reactant matches. In terms of graphs, a rule is a graph transformation with a dynamic rate that determines how frequently the transformation is applied to each matching subgraph [15].

There are six basic transformations that can be encoded in a BNGL rule: (i) bond addition, (ii) bond deletion, (iii) state change, (iv) molecule synthesis, (v) molecule deletion, and (vi) complex deletion.[1] The transformations are implied by the differences between the reactant and product patterns in a rule. For example, the rule

```
Egf(r) + Egfr(l,r) -> Egf(r!0).Egfr(l!0,r) k
```

matches `Egf` molecules with unbound `r` components and `Egfr` molecules with unbound `l` and `r` components. The rate law is of the mass-action type (by default [6,7]) with a rate constant `k`. When the rule fires a bond is formed between the `r` component of `Egf` and the `l` component of `Egfr`. We refer the reader to Refs. [6,7] for further details regarding the specifics of the BNGL syntax.

S3.2 Anatomy of a BNGL file

BioNetGen models are specified within text files containing the `.bngl` extension. Model elements are specified in blocks delimited by `begin` and `end` tags. The entire model specification is enclosed within a `begin model` / `end model` block and the model specification itself is contained within six additional blocks: `parameters`, `molecule types`, `seed species`, `observables`, `functions`, and `reaction rules`. Though not strictly

[1]Complex *synthesis* is not considered to be an elemental transformation but rather a composite action comprised of molecule syntheses and bond additions. This is in contrast to complex *deletion* which involves breaking bonds and deleting molecules that are complexed with the molecules listed in the reactant pattern but are not part of the pattern themselves. Basically, additional operations are required to delete a complex, which is why we consider it to be an elemental transformation unto itself.

required, it is suggested that these blocks appear in this order. Molecule types, reaction rules, and observables have been discussed above. Values of rate parameters, initial populations of species, etc., are listed in the `parameters` block and can be referenced in any subsequent block. All initially-populated species and their population levels are specified in the `seed species` block. These act as the starting point for network generation in BioNetGen and define the initial system state for any subsequent simulations. Arbitrary functions of observables can be defined in the `functions` block and used in place of rate constants in the `reaction rules` block. Two types of functions can be defined: global and local [17,18]. Local functions take as an argument a reference to a complex or molecule and evaluate observables over that complex/molecule only. In contrast, global functions evaluate observables over the entire domain.

Any actions to be performed on the model must be listed after the model specification (i.e., the `end model` directive). Common actions include generating a network by iterative application of the rule set to the set of initial seed species, simulating a network (either deterministically or stochastically), performing a "network-free" simulation, changing or resetting the values of species populations or rate parameters, reading in a previously-generated network, and exporting the model and/or network in various file formats (e.g., SBML [19], M-file [20]). If multiple actions are listed in sequence then each is performed using the final system state from the previous action as the initial state for the current action (unless the system is explicitly reset to the initial conditions). For example, one might run an initial equilibration simulation in the absence of ligand, then add a dose of ligand and run a second simulation to investigate the dose-response behavior of the system. We refer the reader to Refs. [6–8] for further details regarding model specification and action syntax in BioNetGen.

S3.3 Generating and simulating an HPP model with BioNetGen/NFsim

From a BioNetGen user's perspective, once a set of species has been selected for lumping, converting a standard BNGL model into an HPP model requires only two additional steps. First, the population-mapping rules (see "Population species and population-mapping rules" in the main text) must be specified within a `population maps` block, either within the same BNGL file (for convenience) or a separate configuration file (to preserve the integrity of the original model). Note that this is the only place where the population species should be specified, they should not be included in the `molecule types` or `seed species` blocks (Sec. S3.2). The lumping rate constant should be set to a large value (NFsim does not currently support infinite rates) and can be specified either as a number or as a reference to a parameter in the `parameters` block. See Text S3, S6, S9, S12, and S16 for examples of HPP configuration files.

Next, the `generate_hybrid_model()` action must be invoked after the model specification. Arguments are passed to `generate_hybrid_model()` in a comma-separated list enclosed by curly braces and each argument/value pair has the form `argument=>value`. Allowed arguments are:

- `overwrite=>0/1`: Disable/enable automatic overwriting of any existing hybrid model (default: `0`).
- `execute=>0/1`: Disable/enable execution of actions after generation of hybrid model (default: `0`).
- `verbose=>0/1`: Disable/enable verbose output during hybrid model generation (default: `0`).
- `prefix=>"string"`: Set the HPP model prefix to value of `"string"` (default: `""`).
- `suffix=>"string"`: Set the HPP model suffix to value of `"string"` (default: `"hpp"`).
- `actions=>["action1","action2",...]`: Define a list of actions (as strings) to be included at the end of the HPP model file.
- `safe=>0/1`: Disable/enable 'safe' mode (default: `0`); *BioNetGen 2.2.4 or later.*

A call to `generate_hybrid_model()` instructs BioNetGen to generate and write the HPP model to the file `[model]_[suffix].bngl`, where `[model]` is the original BNGL filename and `[suffix]` is `"hpp"` by default and can be changed by the user using the `suffix` argument.

In generating the HPP model, BioNetGen automatically appends all of the population species defined in the `population maps` block to the original `molecule types` block, each followed by a `population` keyword to distinguish it as a population type. The `seed species` block is modified by replacing any structured

species selected for lumping with their population species counterparts. The model observables are also modified to account for the introduction of the population species, which is straightforward. Observables are collections of one or more patterns [6, 7]. Therefore, each structured species selected for lumping is matched against each pattern in each observable and if a match is found then the associated population species is added to the observable list. Finally, PNE is performed to obtain the expanded rule set, to which the population-mapping rules are appended in the `reaction rules` block. A before-and-after illustration of this process can be seen by comparing the original (Text S2) and partially-expanded (Text S3) versions of the simple receptor activation model considered in "Partial network expansion" and Figs. 2 and 3 of the main text.

If `execute=>1` is passed to `generate_hybrid_model()`, BioNetGen will additionally perform any actions on the HPP model that are listed in the `actions` argument. For example, a NFsim simulation can be run through BioNetGen by calling the `simulate_nf()` action [7,8], e.g.,

```
generate_hybrid_model({execute=>1,actions=>["simulate_nf({t_end=>10,n_steps=>100})"]}).
```

Alternatively, a XML encoding of the HPP model can be obtained by invoking the `writeXML()` action:

```
generate_hybrid_model({execute=>1,actions=>["writeXML()"]}).
```

This allows NFsim simulations to be run in standalone mode (see Refs. [17,18]). Note that NFsim version 1.11 or later is required to run HPP simulations.

S4 BioNetGen graph formalism and the partial network expansion algorithm

This section presents the graph-theoretic formalism of rule-based modeling in BNGL. This material is adapted from a previous graph-based description of BNGL by Blinov et al. [15] and also draws inspiration from the κ-calculus graph formalism of Winskel and co-workers [21]. The formalism of Blinov et al. is oriented towards a population-based approach. Thus, the terminology of the present work diverges to some degree to support a particle-based perspective. We begin in Sec. S4.1 by summarizing the graph-theoretic formalism of BNGL. We then build upon this material in Sec. S4.2 to provide a formal basis for the partial network expansion (PNE) algorithm, complete with pseudocode.

S4.1 Graph-theoretic BNGL

S4.1.1 Pattern graphs

Pattern graphs are objects that describe substructures, or "motifs," within molecular complexes. Similar to the concept of a functional group in organic chemistry, a pattern graph specifies a molecular motif, or *moiety*, that participates in a characteristic class of reactions. Any instance of a pattern graph within a complex will participate in its characteristic reaction without regard to the composition and structure of the surrounding complex.

Definition 1 (Pattern Graph). *Given a countable index set $\mathbb{I}$, a set of type labels Ω_T, and a set of state labels Ω_S, a pattern graph is defined by the tuple*

$$P \models (M \subset \mathbb{I},\ C \subset M \times \mathbb{I},\ B \subset C \times C_{\pm},\ \texttt{type} : M \cup C \to \Omega_T,\ \texttt{state} : C \to \Omega_S \cup \{\emptyset, ?\}),$$

where M is the set of **molecules**, *C is the set of* **components**, *B is the set of* **component bonds** *(edges), $C_{\pm} = C \cup \{+, -\}$ is the set of components augmented with the* **non-specific bond** *wildcard '+' and the* **null bond** *'−',* `type` *is a function that assigns a type label to each molecule and component, and* `state` *is a function that assigns a state label to each component ('$\emptyset$' is the* **null state** *and '?' is the* **unspecified state** *wildcard). Furthermore, P satisfies the following: (i) at most one bond per component; (ii) bonds are undirected; (iii) no self bonds. The set of all pattern graphs is denoted by $\mathbb{P}$.*

Each component in C is contained by a unique molecule in M. If $c = (m, n) \in C$, then component c is *contained* by molecule m. Components can be thought of as the nodes of the pattern graph, bonds as edges between nodes, and molecules as groups of components.

Definition 2 (Connected Pattern Graph)**.** *A pattern graph P is* **connected** *if every pair of molecules in the pattern graph is connected by a sequence of component bonds.*

Example: Representation of a BNGL pattern as a pattern graph

Consider the BNGL pattern

$$\texttt{L(r!1).R(l!1,a}\sim\texttt{P)}.$$

Let us represent this pattern formally as a pattern graph. We begin by assigning a unique index to each molecule (from left to right) and a unique ordered pair of indices to each component (also from left to right), where the first element of the pair is the index of the molecule containing the component:

$$\texttt{L}_1\texttt{(r}_{(1,1)}\texttt{!1).R}_2\texttt{(l}_{(2,1)}\texttt{!1,a}_{(2,2)}\sim\texttt{P)}.$$

Now we can write the corresponding pattern graph tuple $P = (M, C, B, \texttt{type}, \texttt{state})$, where

$$\begin{array}{ccl} M & = & \{\ 1,\ 2\ \} \\ C & = & \{\ (1,1),\ (2,1),\ (2,2)\ \} \\ B & = & \{\ \big((1,1),(2,1)\big),\big((2,1),(1,1)\big),\big((2,2),-\big)\ \} \\ \texttt{type} & = & \{\ 1 \mapsto \text{``L''},\ 2 \mapsto \text{``R''},\ (1,1) \mapsto \text{``r''},\ (2,1) \mapsto \text{``l''},\ (2,2) \mapsto \text{``a''}\ \} \\ \texttt{state} & = & \{\ (1,1) \mapsto \emptyset,\ (2,1) \mapsto \emptyset,\ (2,2) \mapsto \text{``P''}\ \} \end{array}$$

Note that the bond from component `r` to component `l` appears in B twice, once for each ordering of the components. This is simply a convention for indicating that the bond is undirected. It is also clear that P is a connected pattern graph because molecules `L` and `R` are connected through this bond.

S4.1.2 Complex graphs and ensembles of complexes

A complex graph, or simply a *complex*, is a pattern graph that defines an assembly of connected molecules with a precise configuration of bonds and states. While a general pattern graph describes parts of a molecular complex, a complex graph describes a complete instance of a molecular complex. In other words, there is no ambiguity, such as unspecified state ('`?`') or non-specific bond ('`+`') wildcards.

Definition 3 (Complex Graph)**.** *A complex graph is a connected pattern graph where: (i) every component state is defined (no state wildcards); (ii) every component has a null bond or a proper bond (no bond wildcards).*

The state of a model system is described by an ensemble of complexes.

Definition 4 (Ensemble of Complexes)**.** *An ensemble of complexes is a finite set of disjoint complexes.*

Intuitively, an ensemble of complexes is a "particle-based" representation of a system, where each particle corresponds to a distinct molecule in one of the complexes.

S4.1.3 Pattern graph isomorphism and species

It is useful to define the notion of equivalence of pattern graphs. We will say that two pattern graphs are equivalent, or *isomorphic*, if they have the same composition and structure.

Definition 5 (Pattern Graph Isomorphism)**.** *Pattern graphs x and y are* **isomorphic***, written $x \cong y$, if there exists a* **one-to-one and onto** *map ϕ of molecules and components in x to those in y that preserves the following properties: (i) graph structure; (ii) component containership; (iii) type; (iv) states. A mapping with these properties is called an* **isomorphism***.*

Since isomorphism is an equivalence relation on the set of complexes, we can partition any ensemble into classes of isomorphic complexes, which we call *species*. This partition is useful because all complexes within a class have the same composition and structure and, therefore, each member has the same reaction motifs and propensities to participate in reaction rules.

Definition 6 (Species)**.** *Given a complex graph y, the species $[y]$ is the set of all complexes that are isomorphic to y. The set of all species is denoted by $\mathbb{S}$.*

Though $[y]$ is properly a set, it can be represented by any complex $y' \in [y]$.

If X is an ensemble of complexes, then a similar but distinct concept is that of the species $[y]$ *with respect to X*, written $[y|X]$. This is the subset of complexes in X that are isomorphic to y, i.e., $\{x \in X \mid x \cong y\}$. The *population* of a species with respect to an ensemble X is the number of complexes in $[y|X]$, i.e., $\rho([y|X]) = |\{x \in X \mid x \cong y\}|$. Thus, a species with respect to an ensemble can be represented by a complex and a population attribute: $[y|X] \equiv (y, \rho([y|X]))$. The set of all species with a population attribute is denoted $\mathbb{S}^\dagger = \mathbb{S} \times \mathbb{N}_{\geq 0}$. Under this notation, an ensemble of complexes X can be represented by a set of species with population counters,

$$S(X) = \{(s, \rho([s|X])) : s \in X/\!\cong\},$$

where $X/\cong$ is the quotient set of X by $\cong$.

We may choose to dispense with the reference to X entirely and directly represent a system as a *species ensemble.*

Definition 7 (Species Ensemble)**.** *A species ensemble S is a set of species with population counters. More precisely,*

$$S \subset \{(s, N_s) \mid s \in \mathbb{S},\ N_s \in \mathbb{N}_{\geq 0}\}\,.$$

If $(s, N_s) \in S$, then we will abuse notation slightly and write $s \in S$ and define $\rho(s) = N_s$. In contrast to an ensemble of complexes, a species ensemble is a "population-based" representation of a system since complexes belonging to the same species are lumped together into a single object.

S4.1.4 Embeddings of pattern graphs

An embedding, or "pattern match," is a map from a pattern to an instance of that pattern within a complex or, more generally, another pattern.

Definition 8 (Embedding)**.** *Given patterns p and q, an* **embedding** *of p in q is a* **one-to-one** *mapping ϕ from the molecules and components of p into those of q that preserves the following properties: (i) graph structure* **compatibility***; (ii) component containership; (iii) type; (iv) state* **compatibility***. The non-specific bond* '`+`' *is compatible with any bond except the null bond* '`-`'*. The unspecified state wildcard* '`?`' *is compatible with any state.*

An embedding of pattern p in pattern q is denoted by $\phi_{p,q}$. If X is an ensemble of complexes, then $\texttt{emb}(p, X)$ is the set of all embeddings of pattern p into the complexes of X. Similarly, the set of embeddings from pattern p into *any* other pattern graph is denoted by $\texttt{emb}(p, \mathbb{P})$. Embeddings of a pattern graph into a species, e.g., $\phi \in \texttt{emb}(p, s)$, where $s \in \mathbb{S}$, should be interpreted as embeddings into the pattern graph representing the species.

It is often useful to refer to the pattern graph or species that contains the specific instance of an embedding. We call this the *target* of the embedding.

Definition 9 (Target)**.** *Given an embedding ϕ, the* **target** *of ϕ, written $\texttt{targ}(\phi)$, is the pattern graph or species that contains the* **image** *of ϕ.*

S4.1.5 Reaction rules

A reaction rule represents a class of potential transformations on an ensemble of complexes.

Definition 10 (Reaction Rule)**.** *A reaction rule is defined by the tuple*

$$r \models \left((R_v)_{v=1}^{V},\ (P_w)_{w=1}^{W},\ \psi,\ k \right),$$

where V is the number of **reactant** *patterns, W is the number of* **product** *patterns,* (R_v) *are the reactant pattern graphs,* (P_w) *are the product pattern graphs,* ψ *is the* **component correspondence map***, and k is the* **propensity***,*[2] *which may be given by a non-negative real number or function.*

The reactant pattern graphs describe motifs in complexes where a reaction event may occur. The product patterns describe the structure at the site of the motif subsequent to transformation under the action of the rule. The component correspondence map is a one-to-one, partial mapping of molecules and components in (R_v) to those of (P_w) that satisfies several properties (see below). The propensity k is defined such that $k{\cdot}dt$ is the probability for each rule instance (described next) to undergo transformation in the next infinitesimal time interval dt [22, 23]. In addition to a simple non-negative constant, the propensity may be a function of the ensemble of complexes (a "global function"), the target of a reactant pattern embedding (a "local function"), or both (a "composite function") [24].

The component correspondence map, ψ, defines how molecules and components in the reactant patterns correspond to those in the product patterns. The map is partial because a subset of molecules and components may be deleted or synthesized by the action of the rule. If a reactant molecule is not in the domain of ψ, $\texttt{dom}(\psi)$, then the molecule and its components are deleted. Similarly, if a product molecule is not in the image of ψ, $\texttt{im}(\psi)$, then the molecule and its components are synthesized. The differences in the bond and state configurations of corresponding components imply a set of pattern graph transformations. For example, if two components in the reactant patterns do not share a bond edge but the corresponding components in the product patterns do, then a "bond addition" transformation is implied by the rule.

Definition 11 (Component Correspondence Map)**.** *Given reactant patterns* $(R_v)_{v=1}^{V}$ *and product patterns* $(P_w)_{w=1}^{W}$*, a component correspondence map* ψ *is a* **one-to-one, partial** *mapping from the molecules and components of* (R_v) *to those of* (P_w)*,*

$$\psi :\ \bigcup_v (M_{R_v} \cup C_{R_v}) \ \rightarrow\ \bigcup_w (M_{P_w} \cup C_{P_w}),$$

that satisfies the following properties: (i) molecules map to molecules and components map to components; (ii) types are preserved; (iii) molecule substructures are preserved; (iv) no new wildcards appear in the product graphs.

It is useful to define the notion of the "reaction center" of a rule, which is the subset of molecules and components in the reactant patterns that are modified by the action of the rule.

Definition 12 (Reaction Center)**.** *The reaction center of a rule r, written* $\texttt{RC}(r)$*, is the subset of reactant molecules not in the domain of* ψ *(deleted molecules)* **plus** *the subset of reactant components in the domain of* ψ *where the corresponding product component: (i) is bound if the reactant component is unbound; (ii) is unbound if the reactant component is bound; (iii) has a different state than the reactant component.*

Conversely, molecules and components in the reactant patterns of a rule that are *not* within the reaction center are called the "reaction context." In BNGL, the reaction context represents additional conditions necessary for a transformation encoded in a rule to take place. By convention, the reaction context does not contribute to the propensity of a rule, i.e., multiple matches to the context does not increase the value of the propensity (multiple matches to the center *does*). As such, it is necessary to equate embeddings where the images of the reaction center are the same even if the context differs.

[2] The traditional definition of the "propensity" is as the product of a constant, c, and a *combinatorial factor*, $h(\mathbf{X})$, which is a function of the system state $\mathbf{X}$ and represents the number of different ways in which specific instances of the reactant species can react [22, 23]. Here, we are dispensing with the combinatorial factor and using the term "propensity" to refer to a constant *or function* that is analogous to c in the traditional definition. We sometimes refer to this quantity as an "instance rate." Note that from a particle-based perspective, each complex can be thought of as a unique species with a unit population, i.e., $h(\mathbf{X})=1$. Thus, our definition of the propensity is simply a special case of the traditional definition.

Definition 13 (Embedding Equivalence)**.** *Given a rule r, with reaction center* $\texttt{RC}(r)$*, the embeddings* $\phi, \phi' \in \texttt{emb}(R_v, \mathbb{P})$ *are equivalent with respect to* $\texttt{RC}(r)$ *if* $\phi|_{\texttt{RC(r)}} = \phi'|_{\texttt{RC(r)}}$*, where* $\phi|_{\texttt{RC(r)}}$ *is the restriction of the embedding map ϕ to the molecules and components in* $\texttt{RC}(r)$*.*

The equivalence class of embedding ϕ with respect to $\texttt{RC}(r)$ is written $[\phi]$. When the context is clear, we will usually omit reference to $\texttt{RC}(r)$. Furthermore, since the embedding class is represented by any of its members, we will usually omit the square braces. Note that all embeddings in an equivalence class have the same target. The set of all equivalence classes of embeddings of pattern R is denoted by $\texttt{emb}(R, \mathbb{P})/\,\texttt{RC}(r)$.

In order to apply the transformation(s) encoded by a reaction rule, it is necessary to have an embedding of each reactant pattern into a target. A *rule instance* is a collection of embedding classes, one for each reactant pattern, whose targets can be transformed by the action of a rule.

Definition 14 (Rule Instance)**.** *Given reactant patterns* $(R_v)_{v=1}^{V}$*, a rule instance is a tuple of embedding classes* $([\phi_v]) \in \prod_{v=1}^{V} \texttt{emb}(R_v, \mathbb{P})/\,\texttt{RC}(r)$*.*

The targets of a rule instance are denoted by the tuple $\texttt{targ}(([\phi_v])) = \{\texttt{targ}(\phi_v)\}_{v=1}^{V}$. Note that the targets are not necessarily distinct.

The *action* of a rule is defined by an operator that maps rule instances to products. The products are constructed by transforming the embedding targets according to the graph operations implied by the differences between the reactant patterns and the product patterns via the component correspondence map.

Definition 15 (Action of a Rule on a Rule Instance)**.** *Given a rule r with reactant patterns* $(R_v)_{v=1}^{V}$*, the* **action** *of r is given by an operator that maps rule instances to tuples of products,*

$$r[\,]: \prod_{v=1}^{V} \texttt{emb}(R_v, \mathbb{P})/\,\texttt{RC}(r) \;\to\; (\mathbb{P})^{<\infty},$$

where $(\mathbb{P})^{<\infty}$ *is the set of finite sequences of pattern graphs (although sometimes the pattern graph is the representative of a species).*

The details of this operator are beyond the scope of the present work (see Sec. 3.1 of Blinov et al. [15] for a discussion within the context of network generation). It is sufficient for our purposes to simply state that we have a unique operator that maps rule instances to products.

It may seem awkward that the action of a rule maps embeddings to products rather than targets to products. However, this distinction is important because the same targets may be transformed into different products depending on the specific embedding into those targets. Thus, the action of a rule depends on both the targets and the embeddings, but since the targets are implicit in the choice of embeddings it is unnecessary to explicitly mention them in the definition. Also note that the number of products produced by the action of a rule is not necessarily equal to the number of product patterns in the rule. This is generally rectified by a post-action rejection step. For example, during network generation BioNetGen rejects all reactions generated from a rule that violate the product-side molecularity of that rule [6] (see below for further discussion).

If the targets of a rule instance belong to an ensemble of complexes, then we can naturally define the action of the rule on the ensemble. The instance targets are removed from the ensemble and replaced by the products generated by the action of the rule on the instance.

Definition 16 (Action of a Rule on an Ensemble of Complexes)**.** *Given an ensemble of complexes X, a rule r, and a rule instance* $(\phi_v) \in \prod_v \texttt{emb}(R_v, X)/\,\texttt{RC}(r)$*, the action of r on X* **through** (ϕ_v)*, written* $r[(\phi_v)](X)$*, is*

$$X \mapsto (X \setminus \texttt{targ}((\phi_v))) \cup r[(\phi_v)],$$

where $r[(\phi_v)]$ *is the action of r on* (ϕ_v)*.*

The action of a rule on a species ensemble S is defined similarly except that targets and products belonging to the species in S are handled by modification of the population counter rather than set deletion and addition.

Definition 17 (Action of a Rule on a Species Ensemble)**.** *Given a species ensemble S, a rule r, and a rule instance $(\phi_v) \in \prod_v \mathtt{emb}(R_v, S)/\,\mathtt{RC}(r)$, the action of r on S through (ϕ_v) is*

$$S \;\mapsto\; \Big\{(s, N'_s) \mid s \in S,\; N'_s = N_s - \big|\{v \in \{1 \ldots V_r\} \mid \mathtt{targ}(\phi_v) = s\}\big| + \big|\{w \in \{1 \ldots W_r\} \mid p_w = s\}\big|\Big\},$$

where (p_w) are the products of the action $r[(\phi_v)]$.

Given an ensemble of complexes X, the set of all rule instances for rule r in X is defined to be

$$\mathtt{rinst}(r, X) = \prod_{v=1}^{V} \mathtt{emb}(R_v, X)/\,\mathtt{RC}(r).$$

This set, along with the rule action operator $r[\;]$, encapsulates every possible transformation on an ensemble of complexes by a reaction rule, and forms the basis for the NF simulation approach [3, 17, 24]. The set of all rule instances for a species ensemble S is defined analogously.

A few notes about rule instance propensities. The propensity of a rule instance is the probability of the rule acting on the instance in the infinitesimal time interval dt. There are several factors that determine this probability: (i) k_r, the propensity of the *rule*; (ii) the symmetry factor of the rule; (iii) the targets of the rule instance; (iv) the products of the action. The symmetry factor of a rule is a positive constant associated with the intrinsic symmetry of the reactant and product patterns of the rule. Its computation is the beyond the scope of this document (see Blinov et al. [15] for a discussion). The targets of the rule instance influence the propensity in two ways. First, the targets of the embeddings must be distinct complexes, i.e., the set of targets must have the same molecularity as the rule. If the targets are not distinct then the propensity is zero. Second, if the rule propensity is a *local function* (see Sneddon et al. [17]), then the local structure of the target can influence the propensity. The products can also influence the propensity, but only as a post-action rejection. While it can be shown that the products, in an aggregate sense, always contain embeddings of the product patterns, there is no guarantee that the products will have the same molecularity as the product patterns. After the products are constructed by the action of the rule, the molecularity of the products can be compared to that of the rule. If the molecularity is different, then the products are rejected and the targets restored. Since compliance with product molecularity is handled as a rejection step, the propensity of a rule instance is actually an upper bound on the true propensity.

Definition 18 (Propensity of a Rule Instance for an Ensemble of Complexes)**.** *Given a rule r with propensity k_r and symmetry constant s_r, an ensemble of complexes X, and a rule instance (ϕ_v), the propensity of the rule instance is*

$$a\left((\phi_v)\right) = \frac{k_r}{s_r} \prod_{v=1}^{V} \max\left\{0,\; 1 - \sum_{j<v} \delta(\phi_j, \phi_v)\right\},$$

where $\delta(\phi_j, \phi_v) = 1$ if $\mathtt{targ}(\phi_j) = \mathtt{targ}(\phi_v)$ and 0 otherwise.

Similarly, for a species ensemble,

Definition 19 (Propensity of a Rule Instance for a Species Ensemble)**.** *Given a rule r with propensity k_r and symmetry constant s_r, a species ensemble S, and a rule instance (ϕ_v), the propensity of the rule instance is*

$$a\left((\phi_v)\right) = \frac{k_r}{s_r} \prod_{v=1}^{V} \max\left\{0,\; \rho(\mathtt{targ}(\phi_v)) - \sum_{j<v} \delta(\phi_j, \phi_v)\right\}.$$

Note that the propensity of a rule instance for an ensemble of complexes is just a special case of this definition [i.e., $\rho(\mathtt{targ}(\phi_v)) = 1$ if $\mathtt{targ}(\phi_v)$ is a complex $x \in X$].

S4.1.6 BNGL models

An *observable* defines a measurable output of a model system. Observables are composed of a set of pattern graphs, each corresponding to a molecular motif that is detectable by an experimental assay [6].

Definition 20 (Observable)**.** *An observable is defined by the tuple*

$$\mathcal{O} \models \left(\texttt{name}, \texttt{type} \in \{\text{``Molecules''}, \text{``Species''}\}, (P_v)_{v=1}^{V} \right),$$

where `name` *is a label for the observable,* `type` *is a property that determines how the observable is calculated, and* (P_v) *is a tuple of pattern graphs.*

The *population* of an observable is a measure of the number of embeddings of the pattern graphs within the system. The type of the observable, "Molecule" or "Species", determines the method of computing the population (see Faeder et al. [6]). For an ensemble of complexes X, the population of an observable is

$$\rho(\mathcal{O}|X) = \begin{cases} \sum_{v=1}^{V} |\texttt{emb}(P_v, X)| & : \text{ type} = \text{``Molecules''} \\ \sum_{x \in X} H\Big(\sum_{v=1}^{V} |\texttt{emb}(P_v, x)|\Big) & : \text{ type} = \text{``Species''} \end{cases},$$

where $H(z) = 1$ if $z \geq 1$ and 0 otherwise. Similarly, for a species ensemble S,

$$\rho(\mathcal{O}|S) = \begin{cases} \sum_{s \in S} \rho(s) \sum_{v=1}^{V} |\texttt{emb}(P_v, s)| & : \text{ type} = \text{``Molecules''} \\ \sum_{s \in S} \rho(s)\, H\Big(\sum_{v=1}^{V} |\texttt{emb}(P_v, s)|\Big) & : \text{ type} = \text{``Species''} \end{cases}.$$

A BNGL model completely specifies a dynamic system, including the initial system configuration, the reaction rules that govern the dynamics, and a set of observables that define the outputs of the system.

Definition 21 (Model)**.** *A BNGL model is defined by the tuple*

$$\mathcal{M} \models (\texttt{seed\_species}, \texttt{observables}, \texttt{reaction\_rules}),$$

where `seed_species` *is a subset of* $\mathbb{S}^{\dagger}$ *that specifies the initial state of the model system,* `observables` *is a set of observables, and* `reaction_rules` *is a set of reaction rules that govern the dynamics of the system.*

In practice, a BNGL model may also include parameters, molecule types (component-level type graphs), and function definitions [6], but we will omit these here for simplicity. Note that while seed species are always specified by a species ensemble in a model, the representation within a simulator may be as an ensemble of complexes (particle representation), a species ensemble (population representation), or a *hybrid ensemble* (see below).

S4.2 Formal basis for the PNE algorithm

S4.2.1 Hybrid ensembles

HPP simulation requires the notion of a *hybrid ensemble*. A hybrid ensemble is a particle/population representation of a system, since part of the system is represented as individual particles and the remainder as lumped populations.

Definition 22 (Hybrid Ensemble)**.** *A hybrid ensemble is the pair* (X, S)*, where* X *is an ensemble of complexes and* S *is a species ensemble.*

For convenience, if (X, S) is a hybrid ensemble and $x \in X$, then we define $\rho(x) = 1$. The action of a rule on a hybrid ensemble is defined by combining Defs. 16 and 17.

Definition 23 (Action of a Rule on a Hybrid Ensemble). *Given a hybrid ensemble* (X, S)*, a rule* r*, and a rule instance* $(\phi_v) \in \prod_v \mathtt{emb}(R_v, X \cup S)/\,\mathtt{RC}(r)$*, the action of* r *on* (X, S) *through* (ϕ_v) *is*

$$\begin{aligned} X &\mapsto \Big(X \setminus \mathtt{targ}((\phi_v))\Big) \cup \Big(r[(\phi_v)] \setminus S\Big)\,, \\ S &\mapsto \Big\{(s, N'_s) \mid s \in S,\ N'_s = N_s - \big|\{v \in \{1 \dots V_r\} \mid \mathtt{targ}(\phi_v) = s\}\big| + \big|\{w \in \{1 \dots W_r\} \mid p_w = s\}\big|\Big\}\,. \end{aligned}$$

where (p_w) *are the products of the action* $r[(\phi_v)]$*.*

The set of all rule instances for a hybrid ensemble (X, S) is defined analogously to that for an ensemble of complexes,

$$\mathtt{rinst}\big(r, X \cup S\big) = \prod_{v=1}^{V} \mathtt{emb}(R_v, X \cup S)/\,\mathtt{RC}(r).$$

The propensity of a rule instance for a hybrid ensemble is the same as defined in Def. 19. Finally, the population of an observable for a hybrid ensemble (X, S) is just the sum of the observable counts for the complexes and the species, i.e.,

$$\rho(\mathcal{O}|X, S) = \begin{cases} \sum_{v=1}^{V} |\mathtt{emb}(P_v, X)| + \sum_{s \in S} \rho(s) \sum_{v=1}^{V} |\mathtt{emb}(P_v, s)| & : \text{ type} = \text{“Molecules”} \\ \sum_{x \in X} H\Big(\sum_{v=1}^{V} |\mathtt{emb}(P_v, x)|\Big) + \sum_{s \in S} \rho(s)\, H\Big(\sum_{v=1}^{V} |\mathtt{emb}(P_v, s)|\Big) & : \text{ type} = \text{“Species”} \end{cases}.$$

S4.2.2 Child rules and rule restriction

A reaction rule r' is said to be a *child* of reaction rule r if the rule instances of r' are a subset of those of r and the action of r' is equivalent to r on that subset. In formal terms, there must be a rule instance $(\phi_v) \in \prod_v \mathtt{emb}(R_v, R'_v)$ and a tuple of embeddings $(\phi_w) \in \prod_w \mathtt{emb}(P_w, P'_w)$. Furthermore, it must follow that the component correspondence maps commute, i.e. $\phi' \circ \psi(x) = \psi' \circ \phi(x)$ for all $x \in \mathtt{dom}(\psi)$, where ϕ and ϕ' are the unions of the respective embeddings. A child rule can be constructed from the pairing of a rule r with a rule instance (ϕ_v). The child rule has reactant patterns given by the targets of (ϕ_v) and product patterns constructed by the action of the rule on the instance.

Definition 24 (Restriction of a Rule by a Rule Instance). *The* **child rule** *induced by the restriction of rule* r *by a rule instance* (ϕ_v) *is defined to be*

$$r|(\phi_v) = \big(\,\mathtt{targ}((\phi_v))\,,\ r[(\phi_v)],\ \psi',\ k_r\,\big)\,,$$

where $r[(\phi_v)]$ *are the products of the action of* r *on* (ϕ_v) *and* ψ' *is obtained from a* **single pushout** *of* ϕ *and* ψ *(see Refs. [15, 25]). In general, the targets may be species or pattern graphs.*

Given a parent rule p, child rules r and r' are equivalent if the reactant patterns are isomorphic, the product patterns are isomorphic, and the component correspondence maps commute with the isomorphisms.

Definition 25 (Child Rule Equivalence). *Child rules* r *and* r' *are equivalent, written* $r \cong r'$*, if the reactant patterns are isomorphic via mappings* (ϕ_v)*, the product patterns are isomorphic via mappings* (ϕ'_w)*, and the following hold:*

- $\phi(\mathtt{dom}(\psi)) = \mathtt{dom}(\psi')\,,$
- $\forall z \in \mathtt{dom}(\psi) :\ \psi' \circ \phi(z) = \phi' \circ \psi(z),$

where ϕ *and* ϕ' *are the unions of the embedding maps.*

The class of all child rules equivalent to r is denoted by $[r]$. The equivalence relation partitions the set of child rules into classes. If R is a set of child rules, then the set of all equivalence classes in R is denoted $R/\!\cong$. Child rules in the same class can be treated as a single entity with a *multiplicity factor* that ensures that the propensity of the class is equal to the sum of the individual propensities. A proof is beyond the scope of this document.

S4.2.3 Population-mapping rules

A species is "unstructured" if it is represented by a complex consisting of a single molecule that contains no components. Given a set of structured species S and a set of unstructured species U, where the types in U do not overlap with those in S, an "unstructured representation" of the species in S is a one-to-one mapping $\lambda : S \to U$. In the population-adapted NF simulator, complexes belonging to an unstructured species can be treated as a population (a species ensemble) rather than as individual particles (an ensemble of complexes). Thus, structured species can be treated as populations if an unstructured representation is provided *and* the reaction rules are partially expanded so that the dynamics of the unstructured species are equivalent to those of the structured counterpart. This process is accomplished by the PNE algorithm.

Prior to performing PNE, the modeler defines a set of *population-mapping rules*. Each mapping in an unstructured representation implies a population-mapping rule, and conversely a set of population-mapping rules implies an unstructured representation. Formally, a population-mapping rule is a reaction rule that matches complexes belonging to a single species and transforms them into the unstructured representation.

Definition 26 (Population-mapping rule)**.** *Given an unstructured representation $\lambda : S \to U$, a population-mapping rule for species $s \in S$ is a reaction rule*

$$l_s \models \left((s),\, (\lambda(s)),\, \varnothing,\, k_{\text{lump}} \right),$$

where $k_{\text{lump}} \geq 0$ is the "lumping rate constant."

S4.2.4 The PNE algorithm

Network expansion is the process of enumerating all of the specific reactions (at the resolution of species) implied by a set of reaction rules. The rule set produced by the PNE algorithm is a *partial* network expansion in the sense that the reactions are enumerated only with respect to the population species. Interactions among particles and between particles and population species are encoded by a set of child rules derived from the original rule set. Thus, there are three types of reaction rules among the set of children: (i) rules that govern the interaction of particles; (ii) mixed particle-population rules that govern the interaction of particles and population species; (iii) population *reactions* that govern the interactions among population species. Rules of type (i) are just duplicates of the original reaction rules. Rules of type (iii) are proper reactions since each reactant pattern matches exactly one species. Rules of type (ii) and (iii) together encapsulate every possible interaction among the population species and between population species and reactant motifs within particles. In this sense, PNE is a hybrid of network-based and network-free methods: particles interact within an NF framework, populations interact within a network-based framework, and particle-population interactions are handled in a hybrid manner. Pseudocode for the PNE algorithm is presented as Algorithm 1. The method takes as input a BNGL model and a set of population-mapping rules and returns a model with a partially expanded rule set (an HPP model) that is dynamically equivalent to the original model for sufficiently large k_{lump} (or for any k_{lump} if the "exact" option is selected; see line 21 of Algorithm 1).

When the HPP model is loaded into the population-adapted NF simulator, all of the unstructured species in U are represented as a species ensemble (i.e., as populations). The structured species, however, are represented as an ensemble of complexes X (i.e., as particles). Therefore, the system representation is a hybrid ensemble $(X, U^{\dagger})$. The propensities for rule instances are computed according to Def. 19. However, rule instances in which two or more reactant patterns have the same target are given the same propensity as other instances and then rejected in a post-sampling step. Furthermore, at simulation runtime, population-mapping rules serve the practical purpose of matching unlumped particles belonging to a population species, deleting the particle, and incrementing the population counter of the corresponding unstructured representation. Runtime lumping is required since the PNE algorithm is not guaranteed to identify every possible way that particle-particle or particle-population events can yield a product belonging to the set of population species (e.g., through complex dissociation). However, PNE does enumerate every way that a population species can participate in a reaction as a reactant.

Algorithm 1: Partial Network Expansion

input : model // *a BNGL model (Def. 21)*
k_{lump} // *a lumping rate constant ("large" unless "exact" option selected – line 21)*
$\lambda : S \to U$ // *an unstructured representation of the species in S (Sec. S4.2)*
output: hpp_model // *the output model*

// *Transform seed species in S into the unstructured representation*
1 **foreach** $(s', n_{s'}) \in$ model.seed_species **do**
2   **if** $s' \in S$ **then**
3   **add** $(\lambda(s'), n_{s'})$ **to** hpp_model.seed_species
4   **else**
5   **add** $(s', n_{s'})$ **to** hpp_model.seed_species
6 **foreach** $s \in S$ **do**
7   **if** $\nexists (s', n_{s'}) \in$ hpp_model.seed_species *such that* $s' \cong s$ **then**
8   **add** $(\lambda(s), 0)$ **to** hpp_model.seed_species

// *Reformulate observables (Def. 20) in terms of unstructured representation*
9 **foreach** $(\text{name}, \text{type}, (P_v)) \in$ model.observables **do**
10   $(P'_v) = (P_v)$
11   **if** type = "Molecules" **then**
12   **foreach** *pair* $s \in S,\ P_v \in (P_v)$ **do**
13   **add** $|\texttt{emb}(P_v, s)|$ copies of $\lambda(s)$ **to** (P'_v)
14   **else**
15   **foreach** $s \in S$ **do**
16   **if** $\cup_v \texttt{emb}(P_v, s) \neq \varnothing$ **then**
17   **add** $\lambda(s)$ **to** (P'_v)
18   **add** $(\text{name}, \text{type}, (P'_v))$ **to** hpp_model.observables

// *Expand reaction rules (Def. 10) around the species in S*
19 **foreach** $r := \big((R_v),\ (P_w),\ \psi,\ k \big) \in$ model.reaction_rules **do**
   // *1: Gather reactant pattern embeddings*
20   **foreach** $R_v \in (R_v)$ **do**
21   **if** $\nexists s \in S$ *such that* $s \cong R_v$ OR *if "exact" method is desired* **then**
22   **add** $\texttt{id}_{R_v, R_v}$ **to** embeddings[v] // *i.e., add the identity map of the reactant pattern*
23   **add** $\texttt{emb}(R_v, S)/\texttt{RC}(r)$ **to** embeddings[v] // *Def. 13*
   // *2: Construct a child rule for each rule instance in the Cartesian product of embeddings*
24   **foreach** *rule instance* $(\phi_v) \in \prod_{v=1}^{V}$ embeddings[v] **do** // *Def. 14*
25     $\big((R'_v),\ (P'_w),\ \psi',\ k \big) \ := \ r \mid (\phi_v)$ // *Def. 24*
     // *3: Replace instances of structured population species with unstructured counterparts*
26   **foreach** $R'_v \in (R'_v)$ **do**
27   **if** $\exists s \in S$ *such that* $s = R'_v$ **then** // *equality is required!*
28   **substitute** $\lambda(s)$ **for** R'_v
29   **foreach** $P'_w \in (P'_w)$ **do**
30   **if** $\exists s \in S$ *such that* $s \cong P'_w$ **then** // *isomorphism is sufficient here*
31   **substitute** $\lambda(s)$ **for** P'_w
32   **add** $\big((R'_v),\ (P'_w),\ \psi',\ k \big)$ **to** child_rules
   // *Compute equivalence classes of child rules (Def. 25) and add to* hpp_model
33   **add** (child_rules/$\cong$) **to** hpp_model.reaction_rules
// *Add population-mapping rules (Def. 26)*
34 **foreach** $s \in S$ **do**
35   **add** $\big((s),\ (\lambda(s)),\ \varnothing,\ k_{\text{lump}} \big)$ **to** hpp_model.reaction_rules

References

[1] http://aws.amazon.com/ec2/.

[2] Y. Cao, H. Li, and L. Petzold. Efficient formulation of the stochastic simulation algorithm for chemically reacting systems. *J. Chem. Phys.*, 121:4059–4067, 2004.

[3] J. Yang, M. I. Monine, J. R. Faeder, and W. S. Hlavacek. Kinetic Monte Carlo method for rule-based modeling of biochemical networks. *Phys. Rev. E*, 78:031910, 2008.

[4] M. I. Monine, R. G. Posner, P. B. Savage, J. R. Faeder, and W. S. Hlavacek. Modeling multivalent ligand-receptor interactions with steric constraints on configurations of cell-surface receptor aggregates. *Biophys. J.*, 98:48–56, 2010.

[5] J. R. Faeder, M. L. Blinov, B. Goldstein, and W. S. Hlavacek. Rule-based modeling of biochemical networks. *Complexity*, 10:22–41, 2005.

[6] J. R. Faeder, M. L. Blinov, and W. S. Hlavacek. Rule-based modeling of biochemical systems with BioNetGen. In *Methods in Molecular Biology*, volume 500, pages 113–167. Humana Press, Clifton, N.J., 2009.

[7] J. A. P. Sekar and J. R. Faeder. Rule-based modeling of signal transduction: A primer. In *Methods In Molecular Biology*, volume 880, pages 139–218. Humana Press, Clifton, N.J., 2012.

[8] http://www.bionetgen.org.

[9] V. Danos and C. Laneve. Formal molecular biology. *Theor. Comput. Sci.*, 325(1):69–110, 2004.

[10] V. Danos, J. Feret, W. Fontana, R. Harmer, and J. Krivine. Rule-based modelling of cellular signalling. *Lect. Notes Comput. Sci.*, 4703:17–41, 2007.

[11] L. A. Harris, J. S. Hogg, and J. R. Faeder. Compartmental rule-based modeling of biochemical systems. In M. D. Rossetti, R. R. Hill, B. Johansson, A. Dunkin, and R. G. Ingalls, editors, *Proceedings of the 2009 Winter Simulation Conference*, pages 908–919, 2009.

[12] M. L. Blinov, J. R. Faeder, B. Goldstein, and W. S. Hlavacek. A network model of early events in epidermal growth factor receptor signaling that accounts for combinatorial complexity. *Biosystems*, 83:136–151, 2006.

[13] A. G. Batzer, D. Rotin, J. M. Ureña, E. Y. Skolnik, and J. Schlessinger. Hierarchy of binding sites for Grb2 and Shc on the epidermal growth factor receptor. *Mol. Cell Biol.*, 14:5192–5201, 1994.

[14] Y. Okabayashi, Y. Kido, T. Okutani, Y. Sugimoto, K. Sakaguchi, and M. Kasuga. Tyrosines 1148 and 1173 of activated human epidermal growth factor receptors are binding sites of Shc in intact cells. *J. Biol. Chem.*, 269:18674–18678, 1994.

[15] M. L. Blinov, J. Yang, J. R. Faeder, and W. S. Hlavacek. Graph theory for rule-based modeling of biochemical networks. *Lect. Notes Comput. Sci.*, 4230:89–106, 2006.

[16] N. W. Lemons, B. Hu, and W. S. Hlavacek. Hierarchical graphs for rule-based modeling of biochemical systems. *BMC Bioinformatics*, 12:45, 2011.

[17] M. W. Sneddon, J. R. Faeder, and T. Emonet. Efficient modeling, simulation and coarse-graining of biological complexity with NFsim. *Nat. Meth.*, 8:177–183, 2011.

[18] http://www.nfsim.org.

[19] http://sbml.org.

[20] http://www.mathworks.com/products/matlab/.

[21] V. Danos, J. Feret, W. Fontana, R. Harmer, J. Hayman, J. Krivine, C. Thompson-Walsh, and G. Winskel. Graphs, rewriting and causality in rule-based models. *submitted*. `http://www.cl.cam.ac.uk/~jmh93/papers/biomod.pdf`.

[22] D. T. Gillespie. A general method for numerically simulating the stochastic time evolution of coupled chemical reactions. *J. Comput. Phys.*, 22:403–434, 1976.

[23] D. T. Gillespie. Stochastic simulation of chemical kinetics. *Annu. Rev. Phys. Chem.*, 58:35–55, 2007.

[24] M. W. Sneddon. *Overcoming complexity in systems biology modeling and simulation.* PhD thesis, Yale University, 2012.

[25] H. Ehrig, R. Heckel, M. Korff, M. Löwe, L. Ribeiro, A. Wagner, and A. Corradini. Algebraic approaches to graph transformation part II: Single pushout approach and comparison with double pushout approach. In *Handbook of Graph Grammars and Computing by Graph Transformation*, volume 1, pages 247–312. World Scientific, Singapore, 1996.
